# Hyperglycemia enhances arsenic-induced platelet and megakaryocyte activation

**DOI:** 10.1186/s12967-017-1148-1

**Published:** 2017-03-06

**Authors:** Jonathan D. Newman, Christina T. Echagarruga, Yoscar M. Ogando, Emilie Montenont, Yu Chen, Edward A. Fisher, Jeffrey S. Berger

**Affiliations:** 1 Division of Cardiology and the Center for the Prevention of Cardiovascular Disease, Department of Medicine, New York University School of Medicine, TRB rm. 853, New York, NY 10016 USA; 2 Department of Medicine, Marc and Ruti Bell Program in Vascular Biology and Disease, New York University Medical Center, New York, NY USA; 3 Departments of Medicine, Population Health and Environmental Medicine, New York University Medical Center, New York, NY USA

**Keywords:** Atherothrombosis, Platelet activity, Megakaryocyte adhesion, Inorganic arsenic, Diabetes mellitus, Environmental exposures

## Abstract

**Objective:**

Low to moderate inorganic arsenic (iAs) exposure is independently associated with cardiovascular disease (CVD), particularly for patients with diabetes mellitus (DM). The mechanism of increased CVD risk from iAs exposure in DM has not been adequately characterized. We evaluated whether increasing concentrations of glucose enhance the effects of iAs on platelet and megakaryocyte activity, key steps in atherothrombosis.

**Methods:**

Healthy donor whole blood was prepared in a standard fashion and incubated with sodium arsenite in a range from 0 to 10 µM. iAs-induced platelet activation was assessed by platelet receptor CD62P (P-selectin) expression and monocyte-platelet and leukocyte-platelet aggregation (MPA and LPA, respectively) in the presence of increasing sodium arsenite and glucose concentrations. Megakaryocyte (Meg-01) cell adhesion and gene expression was assessed after incubation with or without iAs and increasing concentrations of d-glucose.

**Results:**

Platelet activity markers increased significantly with 10 vs. 0 µM iAs (P < 0.05 for all) and with higher d-glucose concentrations. Platelet activity increased significantly following co incubation of 1 and 5 µM iAs concentrations with hyperglycemic d-glucose (P < 0.01 for both) but not after incubation with euglycemic d-glucose. Megakaryocyte adhesion was more pronounced after co incubation with iAs and hyperglycemic than euglycemic d-glucose, while gene expression increased significantly to iAs only after co incubation with hyperglycemic d-glucose.

**Conclusion:**

We demonstrate that glucose concentrations common in DM potentiate the effect of inorganic arsenic exposure on markers of platelet and megakaryocyte activity. Our results support recent observational cohort data that DM enhances the vasculotoxic effects of arsenic exposure, and suggest that activation of the platelet-megakaryocyte hemostatic axis is a pathway through which inorganic arsenic confers atherothrombotic risk, particularly for patients with DM.

## Background

The adverse cardiovascular and vasculotoxic effects of long-term exposure to high levels of inorganic arsenic in drinking water have been well characterized [1]. Recent studies have demonstrated an increased risk of cardiovascular disease (CVD), ischemic heart disease (IHD) and mortality from low-moderate drinking water inorganic arsenic (iAs) exposure (10–20 µg/L) common in the United States (U.S.), particularly for patients with diabetes mellitus (DM) [2]. Recent prospective cohort study data indicates the vasculotoxicity and cardiovascular disease risk of environmental pollutants, including inorganic arsenic, may be greater for individuals with diabetes [2, 3]. However, the mechanism of this increased risk of environmental exposures for diabetic vasculopathy has not been studied. Pathological and clinical studies consistently demonstrate that platelets play a key role in atherothrombosis [4], and have shown the importance of the platelet-megakaryocyte hemostatic axis for vascular disease and CVD events [5–7]. Patients with DM exhibit increased platelet activity both in vitro and in vivo, and heightened platelet function may contribute to excess macrovascular risk in patients with DM [8]. A previous in vitro study of iAs and atherothrombosis used very high concentrations of sodium arsenite and did not examine the effects of hyperglycemia on thrombotic risk [9]. We examined whether glucose concentrations common in DM potentiate the effects of iAs on in vitro measures of platelet and megakaryocyte adhesion and activity.

## Methods

### Subjects

Whole blood was collected from healthy donors in the fasting state. Subjects were not on any antiplatelet therapy nor did they have any history of cardiovascular disease, metabolic syndrome or DM. All human experiments were performed in accordance with institutional and state guidelines. Phlebotomy was performed after 10 min of quiet rest. Blood was collected following a clean, problem-free venipuncture, using a 21-gauge needle after a 5 cc discard (a tourniquet was used to obtain access and was removed before blood collection). Blood was collected into vacutainer tubes containing 3.2% (0.105 mol/l) sodium citrate for platelet activity measurements. After collection, each tube was gently inverted 3 times and immediately transferred to the laboratory for processing.

### Reagents

Sodium arsenite was dissolved in dH_2_0 for a stock concentration of 1000 µM then added to whole blood at a concentration of up to 10 µM for a total of 30 min, similar to prior studies [10, 11]. Similar procedures were performed to achieve concentrations of 0.1, 1, 5 µM sodium arsenite. d-glucose was dissolved in dH_2_O for a stock concentration of 500 mM then added to whole blood and megakaryocytes at concentrations of 5, 15 or 25 mM to approximate euglycemia (5 mM d-glucose ≈90 mg/dl blood glucose) to a range of hyperglycemia common in DM (15 mM ≈ 270 mg/dl, 25 mM ≈ 450 mg/dl).

### Flow cytometry

To examine the effect of iAs on platelet activity, we first measured platelet activation by assessing platelet P-selectin exposure and the presence of monocyte and lymphocyte platelet aggregates (MPA and LPA, respectively) in whole blood samples. We began with a 10 µM concentration of sodium arsenite used in prior in vitro models with aortic endothelial [10, 11] and vascular smooth muscle cell cultures [12, 13], a concentration 50–75% less than that used in prior studies of arsenic and thrombosis [9]. P-selectin expression (CD62P) is a cell surface marker primarily expressed by activated platelets and involved in platelet adhesion. To identify platelet specific P-selectin, we performed flow cytometry on whole blood with CD42b and CD61 to constitutively expressed platelet glycoproteins 1b (GP1b) and IIIa (GPIIIa), respectively. Flow cytometric analysis was performed using the BD Accuri flow cytometer (C6 Flow Cytometer). Whole blood was incubated in the dark for 30 min at room temperature with APC-conjugated mouse antibody specific for CD42b (glycoprotein Ib) and FITC—conjugated mouse antibody specific for CD62P (P-selectin) (BD Biosciences) before the mean fluorescence intensity of P-selectin–bound antibody per 10,000 events was measured. P-selectin is a component of the alpha granule membrane of resting platelets that is only expressed on the platelet surface membrane after alpha granule secretion. In-vivo circulating degranulated platelets rapidly lose their surface P-selectin, but continue to circulate and function [14]. Monocyte and leukocyte platelet aggregates provide complementary information on in vivo platelet activation; are independently associated with cardiovascular disease events; [15, 16] and were assessed as events positive to markers CD14-APC and CD45-APC, respectively, in addition to platelet marker CD-61. MPAs were defined as events positive to both monocyte markers (CD14-APC [BD Biosciences]) and the platelet marker CD61-FITC (Dako). Monocytes were identified by their staining with CD14-APC and by their characteristic orthogonal light scatter. Monocytes with adherent platelets were identified by CD14-APC positivity. LPAs were defined as events positive to both leukocyte markers (CD45-APC [BD Biosciences]) and the same platelet marker CD61-FITC (Dako). The leukocytes with adherent platelets were identified by CD45-APC positivity. Appropriate color compensation was determined in singly labeled samples and matched nonspecific antibody controls (Mouse IgG1 FITC [BD Biosciences]). For the co-incubation experiments, whole blood was first incubated with 5 and 15 mM d-glucose for 30 min. We then used lower concentrations of sodium arsenite at 0, 0.1, 1 and 5 µM which were added to solution and incubated for an additional 30 min. P-selectin expression with unstimulated and stimulated with thrombin 0.025 IU/ml (Sigma) was then assessed.

### Cell culture and megakaryocyte gene expression

Meg-01 cells were purchased from American Type Culture Collection (VA) and cultured in RPMI-1640 medium supplemented with 10% heat-inactivated fetal bovine serum (FBS), 100 U/ml penicillin, and 100 μg/ml streptomycin (Invitrogen, CA, USA) at 37 °C in a 5% CO_2_ humidified atmosphere, consistent with prior studies [17, 18]. For adhesion assays, 18 mm glass coverslips (Fisher Scientific) coated with collagen (Helena Laboratories, Beaumont, TX, USA) were blocked with 1% BSA in 12-well plates [17, 18]. Meg-01 cells were stained for 10 min with 1 µM DiOC6 (Fisher Scientific), washed and incubated at 2.5 10^5^ cells/ml for 3 h with and without addition of iAs (0, 1, 5 and 10 µM) in presence of 5 or 25 mM d-glucose. After the supernatant was aspirated, adherent cells were gently washed with FBS. For each well, five random fields were captured and area of coverage was quantified using Image J (National Institutes of Health, Bethesda, MD).

Nuclear transcription factor kappa B (NFκB) gene expression was measured because of its roles in inflammation, platelet activation, and arsenic vasculopathy [18–20]. Other genes measured include monocyte chemoattractant protein-1 (CCL2) and CD36 that have also been associated with platelet degranulation, diabetes and inflammation. To measure these genes, total RNA was isolated from Meg-01 cells using the Direct-zol RNA Miniprep kit (ZymoResearch, Irvine, CA, USA) and quantified using a Nanodrop ND-2000 spectrophotometer (Wilmington, DE, USA). RNA was converted to cDNA using the iScript cDNA synthesis kit (BioRad). Gene expression of GAPDH and NFκB1 using the Sso fast Evagreen Supermix (BioRad) was assessed with real-time PCR (iCycler Real-Time Detection System, Eppendorf). The sequences of the NFκB1, CCL2 and CD36 primers used for qRT-PCR were CAGATGGCCCATACCTTCAAA and TTGCAGATTTTGACCTGAGGG, CCCAAAGAAGCTGTGATCTTCA and GCAGATTCTTGGGTTGTGGA, and CTATTGGGAAGGTCACTGCGA and CAGGTCTCCCTTCTTTGCATT, respectively.

### Statistical analysis

All experimental values are represented as mean ± standard error of the mean (SEM). Differences in selected categorical variables between the respective comparison groups were analyzed with the χ^2^ test of statistical significance. Unpaired two-tailed *t* tests and ANOVA were used to examine differences in continuous variables overall and at each time point under study in the different comparison groups. A value of *P* < 0.05 was considered statistically significant.

## Results

**Fig. 1:**
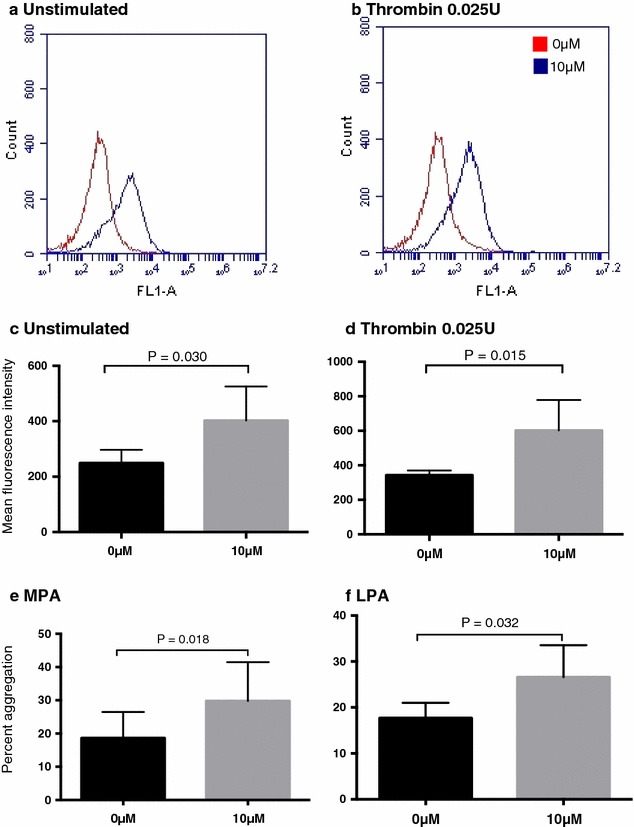
Unstimulated (**a**, **c**) and thrombin-stimulated (**b**, **d**) platelet activitation by flow cytometry (**a**, **b**), mean fluorescence intensity (**c**, **d**) and platelet aggregation (**e**, **f**) to 0 and 10 μM sodium arsenite

We first examined the effect of a 10 µM iAs concentration used previously in endothelial and smooth muscle cell culture to assess the effects of inorganic arsenic exposure [10–13]. There was a clear increase in platelets expressing P-selectin by flow cytometry following incubation with 10 µM iAs (Fig. 1a, b). Compared to 0 µM iAs, the mean fluorescence intensity of P-selectin expression increased significantly after incubation with 10 µM iAs for both unstimulated and thrombin-stimulated platelets (Fig. 1c, d). We subsequently examined the effect of iAs on monocyte and leukocyte platelet aggregation (MPA and LPA, respectively) a different measure of platelet activity predictive of CVD events [15]. Compared to 0 µM, incubation with 10 µM iAs significantly increased both MPA and LPA (Fig. 1e, f). These experiments demonstrate that sodium arsenite concentrations below those used in prior studies of platelet activation have significant effects on multiple measures of platelet activity [9, 21].

**Fig. 2:**
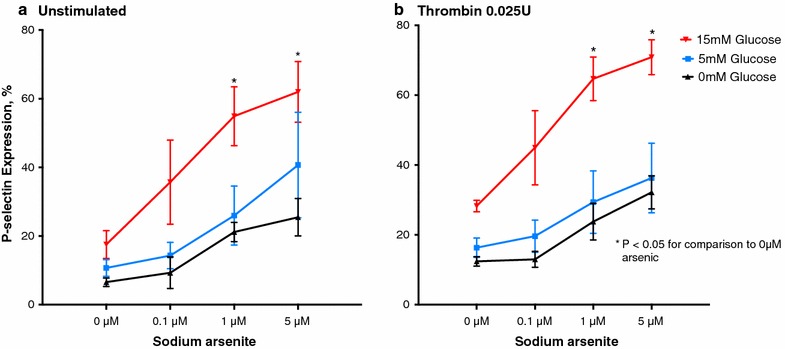
Unstimulated (**a**) and thrombin-stimulated (**b**) percent p-selectin expression to 5 and 15 mM glucose with increasing concentrations of sodium arsenite

Consistent with prior data [8], platelet activity increased with increasing d-glucose concentrations (Fig. 2a, b). To investigate whether glucose and arsenic had a synergistic effect on platelet activation, we coincubated euglycemic (5 mM ≈ 90 mg/dl) and hyperglycemic (15 mM ≈ 270 mg/dl) concentrations of d-glucose with lower concentrations of sodium arsenite than used to demonstrate platelet activation without glucose coincubation. After incubation at hyperglycemic conditions, exposure to 0.1, 1 and 5 µM sodium arsenite led to marked increases in platelet activation. In contrast, these sodium arsenite concentrations did not potentiate platelet activation at euglycemic concentrations of d-glucose (Fig. 2a, b).

**Fig. 3:**
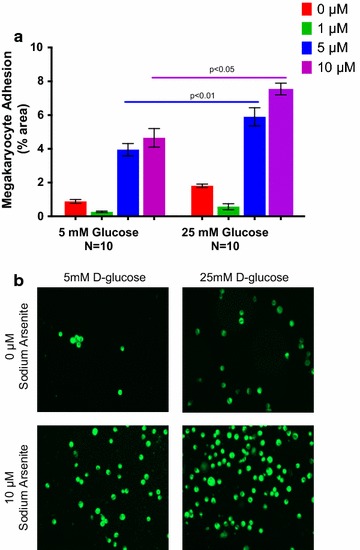
Megakaryocyte adhesion (% area, **a**) and photomicrograph (**b**) to 5 and 25 mM d-glucose with increasing concentrations of sodium arsenite

**Fig. 4:**
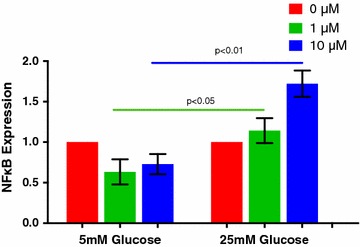
GAPDH Normalized NFκB Gene Expression to 5 and 25 mM d-glucose with increasing concentrations of sodium arsenite

**Fig. 5:**
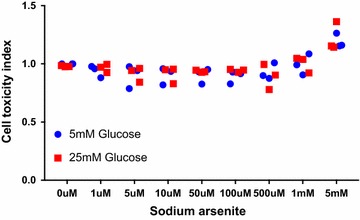
Meg-01 cell toxicity index with 5 and 25 mM d-glucose with increase concentrations of sodium arsenite

Hyperglycemia may induce prothrombotic changes in megakaryocyte function and platelet thrombogenesis [6]. To test whether glucose and iAs also had a synergistic effect on megakaryocyte adhesion, we coincubated megakaryocytes at euglycemic (5 mM) and hyperglycemic (25 mM) concentrations of d-glucose with 0, 1, 5 and 10 mM concentrations of sodium arsenite. Similar to the results observed for platelet activation, exposure to subthreshold sodium arsenite concentrations below 10 µM induced significantly greater megakaryocyte adhesion after incubation with a hyperglycemic compared to a euglycemic concentration of d-glucose (Fig. 3a, b). Prior studies have demonstrated megakaryocyte nuclear transcription factor kappa B (NFκB) gene expression is an important regulator of inflammation and platelet activation [18, 19], and may also be an important transcriptional factor for the vascular effects of inorganic arsenic exposure [20]. To verify the prothrombotic effect of iAs, we measured the gene expression of NFKB1 in Meg-01 cells. monocyte chemoattractant protein-1 (CCL2) and CD36, genes involved in platelet activation and degranulation [22, 23], were also measured [22, 23]. Following coincubation of hyperglycemic d-glucose with 5 and 10 µM sodium arsenite, Meg-01 cells NFκB1 expression increased significantly compared to coincubation with euglycemic d-glucose (Fig. 4). There were additional non-significant increases in MCP-1 (CCL2) and CD36 (data not shown). No deleterious effects on Meg-01 cell toxicity were observed within the range of concentrations of sodium arsenite used in this study (0–10 µM) up to concentrations 100-fold greater (Appendix, Fig. 5).

## Discussion

There are four primary findings of this report. First, we show for the first time that a concentration of d-glucose common in DM potentiates sodium arsenite-induced platelet activation. Second, we demonstrate that hyperglycemia also potentiates the effects of sodium arsenite on megakaryocyte adhesion, a marker of atherothrombotic risk [18]. Third, we demonstrate that lower concentrations of sodium arsenite than previously studied are associated with increased platelet activation and aggregation. Finally, we show that Meg01 NFκB transcription as a marker of megakaryocyte activation increases following exposure to hyperglycemia and sodium arsenite. These findings suggest that alterations in the platelet-megakaryocyte axis may be a pathway through which exposure to environmental toxicants such as iAs increase CVD risk, particularly for patients with DM.

Despite advances in effective medical therapy to reduce CVD events, nearly 70% of patients with DM will die of CVD [24]. The etiology of this excess CVD risk for DM patients remains unclear. The vasculotoxicity and cardiovascular disease risk of environmental pollutants, including iAs, may be greater for individuals with diabetes [2, 3], and suggests that low-level environmental exposures may be a novel risk factor for CVD risk in DM. Environmental pollutants enhance inflammation and the generation of reactive oxygen species, steps also important in the pathogenesis of diabetic vasculopathy [25]. While prior studies have indicated that environmental exposures increase oxidative stress and platelet activation [26, 27], to our knowledge this is the first report to describe a potential link between diabetic hyperglycemia and enhanced atherothrombotic risk to iAs exposure.

There are a number of pathways of platelet activation shared between hyperglycemia and iAs exposure. Hyperglycemia and diabetes is associated with platelet hyperreactivity, and coupled with enhanced levels of thromboxane, may partially explain increases in cardiovascular disease morbidity and mortality seen among patients with DM [8]. High levels of drinking water inorganic arsenic (500 ppb) increase platelet thromboxane formation and adhesion protein expression [28]. Other synergistic pathways between hyperglycemia and iAs exposure include increases in aldose reductase activity and oxidative stress signaling. During hyperglycemia aldose reductase activity increases significantly, leading to abnormal activation of the polyol pathway and enhanced oxidative and osmotic stress [8]. In turn aldose reductase increases thromboxane formation and platelet activation [8]. Inorganic arsenic has also been shown to increase aldose reductase activity [29]. Taken together enhanced aldose reductase activity and thromboxane generation may represent a synergistic pathway of thrombotic risk for both hyperglycemia and inorganic arsenic exposure. Platelet and endothelial mitochondrial function may be another synergistic pathway of risk for iAs exposure in diabetes. Recent studies have indicated the importance of platelet mitochondrial function in cardiovascular disease [30], and have suggested that alterations in platelet mitochondrial function may increase the risk of diabetic atherothrombosis [31]. Inorganic arsenic has also been shown to alter endothelial cell mitochondrial function [13]. Future studies might consider the synergy of inorganic arsenic exposure and diabetes on mitochondrial function in platelets and vascular endothelium as novel pathways of cardiovascular disease risk.

Strengths of the current study include the use of multiple validated measures of the platelet-megakaryocyte axis associated with incident CVD; use of sodium arsenite concentrations below those used in previous models of iAs-induced atherothrombosis; and an investigation of the synergy between hyperglycemia and iAs exposure on atherothrombotic risk. Although we used a lower sodium arsenite concentration than previous atherothrombosis studies [9, 21], we recognize the concentrations of sodium arsenite used may not correspond to current levels of iAs exposure in the U.S. Future studies should further investigate effects at very low concentrations corresponding to levels more prevalent in human populations. The discrepancy between exposure levels relevant to naturally contaminated drinking water and in vitro concentrations of sodium arsenite may reflect the lack of an accepted biomarker of internal iAs dose. Other limitations include the use of in vitro models and the inability to model in vivo differences in hyperglycemia and insulin resistance seen in type 1 and 2 diabetes. Further study is also needed to better estimate internal inorganic arsenic dose relevant for in vitro modeling; to examine the effect of environmental exposures on the platelet-megakaryocyte axis across the spectrum of diabetes control; and to study the effects of iAs and hyperglycemia on mitochondrial function in platelets and other relevant systems. Treatment studies could consider the use of aldose-reductase inhibitors to attenuate platelet activation and megakaryocyte adhesion [8].

## Conclusion

Our findings suggest that increased platelet activation and megakaryocyte adhesion may be pathways through which hyperglycemia in DM can enhance the vasculotoxicity of inorganic arsenic exposure. While intensive glycemic control has failed to significantly reduce macrovascular risk in DM, exposure to environmental toxicants such as inorganic arsenic may represent a novel class of modifiable CVD risk factors, particularly for patients with diabetes. Future studies should investigate platelet activation in patients with and without diabetes, at varying levels of glycemic control, following exposure to environmentally relevant concentrations of inorganic arsenic and other environmental exposures.

## Appendix

See Fig. 5.

## Abbreviations

iAs: inorganic arsenic
CVD: cardiovascular disease
DM: diabetes mellitus
MPA: monocyte-platelet aggregation
LPA: leukocyte-platelet aggregation
Meg-01: megakaryocyte
NFκB: nuclear transcription factor kappa B
FBS: fetal bovine serum
RNA: ribonucleic acid
GAPDH: glyceraldehyde 3-phosphate dehydrogenase
PCR: polymerase chain reaction
SEM: standard error of the mean
ANOVA: analysis of variance
U.S.: United States
